# Study of a genetic collection of strawberry (Fragaria L.) for resistance to powdery mildew

**DOI:** 10.18699/vjgb-24-19

**Published:** 2024-04

**Authors:** A.S. Lyzhin, I.V. Luk’yanchuk

**Affiliations:** I.V. Michurin Federal Scientific Center, Michurinsk, Russia; I.V. Michurin Federal Scientific Center, Michurinsk, Russia

**Keywords:** strawberry, powdery mildew, resistance, molecular markers, QTL, земляника, мучнистая роса, устойчивость, молекулярные маркеры, QTL

## Abstract

Powdery mildew (Sphaerotheca macularis Mag. (syn. Podosphaera aphanis Wallr.)) is a dangerous disease of strawberry (Fragaria L.). The resistance of strawberry to powdery mildew is controlled polygenically. Several genetic loci with a large contribution to disease resistance have been identified in various strawberry varieties. Diagnostic DNA markers have been developed for QTL 08 To-f. They showed a high level of reliable gene detection in mapping populations. The purpose of this study was assessment of a strawberry genetic collection for resistance to powdery mildew and identification of promising strawberry forms for breeding for resistance to S. macularis. The objects of the study were wild species of the genus Fragaria L., varieties and selected seedlings of strawberry (Fragaria × ananassa Duch.) created in the I.V. Michurin Federal Scientific Center, and strawberry varieties introduced from various ecological and geographical regions. To identify QTL 08 To-f, DNA markers IB535110 and IB533828 were used. Locus 08 To-f was detected in 23.2 % of the analyzed strawberry genotypes, including wild species F. moschata and F. orientalis, strawberry varieties of Russian breeding (Bylinnaya and Sudarushka) and foreign breeding (Florence, Korona, Malwina, Ostara, Polka and Red Gauntlet). The correlation between the presence of markers IB535110 and IB533828 and phenotypic resistance (powdery mildew effect on strawberry plants is absent) was 0.649. The determination coefficient (R2 ) showing the contribution of the studied locus to the manifestation of the trait was 0.421, that is, in 42.1 % of cases resistance was explained by the presence of QTL 08 To-f, and in 57.9 % of cases, by other genetic factors. All strawberry genotypes with locus 08 To-f were characterized by high field resistance to S. macularis in the conditions of Michurinsk, Tambov region. Thus, locus 08 To-f is promising for conferring resistance on local powdery mildew races, and markers IB535110 and IB533828 can be used in marker-assisted breeding programs to create powdery mildewresistant strawberry genotypes.

## Abstract (ru)

Мучнистая роса (Sphaerotheca macularis Mag. (син. Podosphaera aphanis Wallr.)) – опасное заболевание земляники (Fragaria L.). Устойчивость земляники к мучнистой росе контролируется полигенно. У различных сортов земляники садовой идентифицировано несколько генетических локусов (QTL) с большим вкладом в устойчивость к болезни. Для QTL 08 To-f разработаны диагностические ДНК-маркеры, показавшие высокий уровень надежности выявления гена в картирующих популяциях. Цель настоящего исследования – изучение генетической коллекции земляники по устойчивости к мучнистой росе и идентификация перспективных для селекции на устойчивость к S. macularis форм. Объектами исследования были дикорастущие виды рода Fragaria L., сорта и отборные сеянцы земляники садовой (F. × ananassa Duch.) селекции Федерального научного центра им. И.В. Мичурина, а также формы, интродуцированные из различных эколого-географических регионов. Для идентификации QTL 08 To-f использовали маркеры IB535110 и IB533828. Локус 08 To-f обнаружен у 23.2 % генотипов земляники, в том числе у дикорастущих видов F. moschata и F. orientalis, сортов земляники садовой отечественной (Былинная, Сударушка) и зарубежной (Florence, Korona, Malwina, Ostara, Polka, Red Gauntlet) селекции. Степень корреляции между наличием маркеров IB535110 и IB533828 и фенотипической устойчивостью (признаки поражения мучнистой росой отсутствуют) составила 0.649. Коэффициент детерминации (R2 ), показывающий вклад изучаемого локуса в формирование признака, равен 0.421, т. е. в 42.1 % случаев устойчивость определяется наличием QTL 08 To- f, тогда как в 57.9 % случаев влияние оказывают факторы внешней среды. Все генотипы земляники с локусом 08 To-f характеризуются высокой полевой устойчивостью к S. macularis в условиях г. Мичуринска Тамбовской области. Таким образом, локус 08 To-f является перспективным для придания устойчивости к местным расам мучнистой росы, а маркеры IB535110 и IB533828 могут быть использованы в программах маркер-опосредованной селекции по созданию устойчивых к мучнистой росе генотипов земляники

## Introduction

Powdery mildew is a dangerous disease of strawberry. The causative agent of powdery mildew is the obligate biotrophic fungus Sphaerotheca macularis Mag. (syn. Podosphaera aphanis Wallr.). The greatest harmful effect to plantings is caused by the conidial stage of the pathogen – Oidium fragariae Harz. (Holod, Semenova, 2014; Tapia et al., 2021). Powdery mildew affects all above-ground plant organs. The infection manifests itself in the form of a white powdery coating of mycelium and conidia of the fungus. Severely affected strawberry leaves curl upward in the shape of a boat, infected peduncles form deformed fruits, tendrils and young rosettes are stunted in growth and subsequently die (Kennedy et al., 2013; Stolnikova, Kolesnikova, 2017). Strawberry yield losses from powdery mildew can exceed 60 % (Nelson et al., 1995; Lifshitz et al., 2007).

Powdery mildew especially affects strawberry plantings in protected soil (greenhouses, hotbeds, tunnels) due to favorable conditions for the development of the pathogen – elevated temperature and humidity (Sylla et al., 2013; Tapia et al., 2021). The development of the pathogen in strawberry plantings is facilitated by warm weather (temperature 18–24 °C) and high air humidity (about 100 %). The decline of the disease is observed when the air is excessively dry or there is an abundance of precipitation (it washes away the pathogen spores and improves the condition of the plants), as well as at temperatures below 15 °C and above 30 °C (Zubov, 1990, 2004). An earlier manifestation of the disease was noted in the spring after warm and snowy winters. In frosty and snowless winters, the main supply of infection dies, and late and weak development of the pathogen is observed (Govorova, Govorov, 2004).

Control of the spread of S. macularis in strawberry plantations is ensured primarily by the use of contact (sulfur) and systemic (captan, benomyl) fungicides (Bajpai et al., 2019; Palmer, Holmes, 2021). However, the active use of chemical plant protection products contradicts the global trend in the development of agriculture – its biologization and ecologization (Zhuchenko, 2009; Gorgitano, Pirilli, 2016). In addition, S. macularis is characterized by a high ability to develop resistance to fungicides (Carisse, Bouchard, 2010; Sombardier et al., 2010). In this regard, a promising direction for increasing resistance to powdery mildew is to identify from the existing assortment and create new strawberry varieties with genetically determined resistance to S. macularis

Resistance of strawberry varieties to powdery mildew is controlled polygenically. The formation of the “powdery mildew resistance” trait depending on the genotype is influenced by both additive (the summed influence of alleles of one gene or several non-allelic genes expressed equally) and non-additive (interaction of alleles of a gene or non-allelic genes according to the type of dominance, overdominance and epistasis due to different levels of gene expression, and as a result one allele of a gene or gene suppressing another) gene effects. The heritability of the “powdery mildew resistance” trait in the strawberry hybrid offspring, according to estimates by various authors, ranges from medium to high (H 2 = 0.44–0.94). This indicates prospects for increasing strawberry resistance to powdery mildew using breeding (Kennedy et al., 2014). Analysis of the inheritance of resistance to S. macularis in strawberry hybrid combinations shows continuous variability of hybrids from resistant to susceptible forms. In a number of strawberry combinations, transgression (additive genetic effects) may occur, leading to the appearance of seedlings that are superior in powdery mildew resistance to the parent forms. Additive effects, as reported by a number of authors (Zubov, 2004; Kennedy et al., 2014), play a major role in the formation of strawberry resistance to powdery mildew.

Some initial strawberry forms (wild species F. orientalis, F. moschata and F. ovalis, and interspecific hybrids 298- 22-19-21 (FB2 F. orientalis, F. moschata, F. × ananassa), 297-22-124, 297-28-84 (FB1 F. orientalis, F. × ananassa) and 778-7 (FB2 F. ovalis, F. × ananassa)) transfer a high level of powdery mildew resistance to a large number of hybrid forms, regardless of the combination of crossing (non-additive genetic effects). The predominance of non-additive gene effects makes it possible to identify donors of strawberry resistance to powdery mildew (Zubov, 2004; Davik, Honne, 2005).

In recent years, several major quantitative trait loci (QTLs) for strawberry resistance to powdery mildew have also been identified. However, they were characteristic only of specific crossing combinations and their manifestation varied depending on prevailing weather conditions. Thus, six QTLs were identified in the hybrid combination Emily × Fenella, and five QTLs were identified in the hybrid combination Red Gauntlet × Hapil. The most stable QTLs include the FaRPa1C (Emily × Fenella) and FaRPa6D2 (Red Gauntlet × Hapil) loci. At the same time, validation of the identified QTLs in a genetically diverse sample of strawberry varieties and forms showed the high uniqueness of the identified powdery mildew resistance loci and their almost complete absence in other genotypes, which limits the possibilities of their use in strawberry breeding (Cockerton et al., 2018).

In the hybrid combination Sonata × Babett, three loci of resistance to powdery mildew (FxaPMR5b, FxaPMR7A and FxaPMR7X2) were identified. Of these, one QTL (FxaPMR7A) was identified when strawberry plants were cultivated in a greenhouse, and two QTLs (FxaPMR5b and FxaPMR7X2) were identified in strawberry plants of open ground (Sargent et al., 2019). However, diagnostic DNA markers for these loci have not been developed, which prevents their use in breeding practice for identifying strawberry forms resistant to S. macularis. In 2020, H. Koishihara and co-authors, based on an analysis of the strawberry hybrid combinations Miyazaki Natsu Haruka × 08 To-f, Miyazaki Natsu Haruka × Ohkimi and 09s E-b45e × Miyazaki Natsu Haruka, identified another QTL (08 To-f ) with a high contribution to the manifestation of resistance to powdery mildew (15.7 %) (Koishihara et al.,2020). The 08 To-f locus size was 6.83 cM. To identify the 08 To-f locus in the strawberry germplasm, diagnostic DNA markers IB535110 and IB533828 were developed. The reliability of identification of strawberry genotypes resistant to powdery mildew using the IB535110 and IB533828 markers in the analyzed crossing combinations was 98.5 %. These markers, according to the authors’ recommendations (Koishihara et al, 2020), can be used for marker-assisted screening of strawberry forms resistant to powdery mildew 

The MLO locus (Mildew Resistance Locus O), encoding genes that affect susceptibility to the pathogen, can also make a certain contribution to the formation of strawberry resistance to S. macularis. Blocking the expression of alleles of these genes or transferring them to a recessive state contributes to the manifestation of strawberry resistance to powdery mildew. 68 MLO sequences have been identified in octoploid strawberry. The most important of them are the FaMLO10, FaMLO17 and FaMLO20 loci (Tapia et al., 2021). In addition, strawberry resistance to powdery mildew is influenced by the TGA family transcription factors (involved in the metabolism of salicylic acid). 11 FaTGA genes were identified in strawberry varieties. The genes FaTGA1, FaTGA2, FaTGA5, FaTGA7, FaTGA8 and FaTGA10 are characterized by the greatest specificity to powdery mildew infection (Feng et al., 2020).

Applied research is also being conducted to develop diagnostic DNA markers to identify powdery mildew-resistant strawberry genotypes. In particular, the scientific group of L.J. Cheng, based on SSR analysis of hybrid seedlings from crossing the varieties Darselect (susceptible to powdery mildew) and Sweet Charlie (resistant to powdery mildew), identified SSR markers FSS50 and FSS121, which showed a close relationship with the presence of phenotypic resistance to S. macularis (Liu et al., 2012). H.-J. Je with co-authors, based on the analysis of the hybrid combination Akihime (susceptible) × Seolhyang (resistant), developed the CAPS marker SP1-Eae I, which allows identifying strawberry genotypes resistant to powdery mildew (Je et al., 2015).

The development of diagnostic DNA markers suitable for use in marker-assisted breeding programs is an important step in increasing the efficiency of selection of pathogen-resistant genotypes and creating new varieties (Whitaker et al., 2012). However, data on the suitability of the identified molecular markers for the analysis of genetically diverse strawberry varieties or wild species of the genus Fragaria L., and the prevalence of resistance loci in the strawberry germplasm, are not provided.

The purpose of this research was to study the strawberry genetic collection according to powdery mildew resistance and identify promising forms for breeding for resistance to S. macularis

## Materials and methods

In this work, we used 43 samples, consisting of wild species of the genus Fragaria L., varieties and selected seedlings of garden strawberry (F. × ananassa Duch.) created in the FSSI “I.V. Michurin FSC”, and strawberry forms originating from different ecological and geographical regions (Table 1).

**Table 1.**
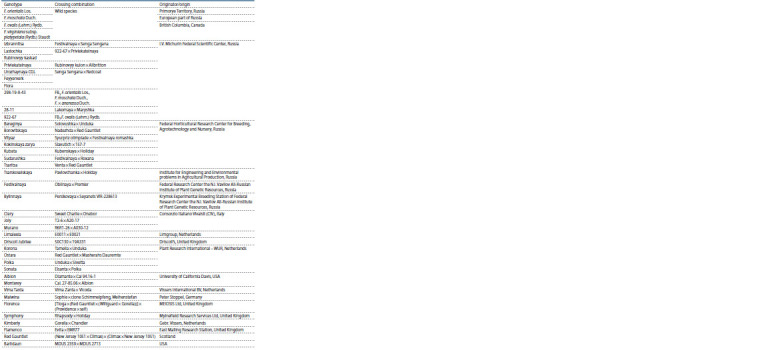
List of wild strawberry species, varieties and selected forms

Weather and climatic conditions of the growing seasons during research (2018–2022) differed from the long-term average. In 2018, average monthly air temperatures exceeded long-term values by 0.7–2.4 °C. 2019 and 2020 have both cooler (July and August 2019, April and May 2020) and hotter months (April, May and June 2019, June, July and September 2020). In 2021 and 2022, the average monthly air temperature exceeded long-term values by 0.6–4.2 °C and 0.8–4.5 °C. Precipitation amount in 2018–2020 was lower than the long-term average values by 13.9–28.7 % and its distribution across the months was quite uniform. In 2021 and 2022, precipitation amount exceeded the long-term average by 2.3 and 16.1 %, respectively. At the same time, precipitation was uneven: there were periods of high humidity (for example, in the 2nd decade of July 2022, 58.8 mm of precipitation fell) and periods of insufficient and weak water availability (August 2021 – 31.3 mm of precipitation, August 2022 – 40.1 mm of precipitation.).

Phenotypic assessment of the powdery mildew resistance of strawberry genotypes was carried out in field conditions against a natural infectious background on a scale from 0 to 5 points, where 0 – no plants affected, 5 – all vegetative organs of the plant are severely affected (Zubov, 1990).

Total genomic DNA was isolated from fresh leaves; extraction was carried out using the CTAB method with modifications described by I.V. Luk’yanchuk et al. (2018).

The QTL 08 To-f was identified with the dominant markers IB535110 and IB533828. DNA marker IB535110 was represented by a 500 bp amplicon. DNA marker IB533828 was represented by an amplicon of about 120 bp. These products were amplified only if the strawberry genotype had the 08 To-f locus (Koishihara et al., 2020).

PCR reactions were performed in 15 μl final volume containing: 20 ng of genomic DNA, 0.2 mM of each dNTP, 2.5 mM MgCl2, 0.2 μM of each primer, 0.2 U of Taq DNA polymerase and 1.5 μl of PCR-buffer (+(NH4)2SO4, –KCI). All components were produced by Thermo Fisher Scientific (USA).

Amplification was performed in a T100 Thermal Cycler (Bio-Rad, USA). PCR conditions were as follows: 94 °C for 1 min, followed by 35 cycles of 94 °C for 30 s, Tm for 30 s, 72 °C for 1 min, and with a final extension of 72 for 5 min. Tm – primers annealing temperature: 35110_v1F/35110_ v1R – 60 °C; 22828_v6F/22828_v6R – 58 °C.

The amplification products were separated on a 2 % agarose gel and visualized by ethidium bromide staining. Gene Ruler 100 bp DNA Ladder (Thermo Fisher Scientific) was used as a molecular weight marker

Experimental data were processed by methods of mathematical statistics using Microsoft Excel 2016 and STATISTICA 6.0. A comparison of the frequency of occurrence of the “powdery mildew resistance” trait in samples of Russian and foreign strawberry varieties was carried out using Student’s t test. The reliability of the results of phytopathological assessment of strawberry genotypes was assessed using two-factor ANOVA. The contribution of a genetic determinant to the formation of a trait was assessed using the heritability coefficient (H 2), calculated using the following formula: H 2 = VG / VP, where VG – genotypic variance, VP – total phenotypic variance. The identification of a statistical relationship between the presence of DNA markers for the 08 To-f locus of powdery mildew resistance and its phenotypic manifestation was carried out by regression analysis using the F-test.

## Results

**Fig. 1.**
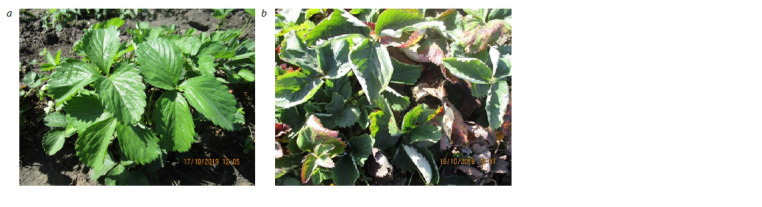
Strawberry plants affected by powdery mildew. a – the Sudarushka variety, no effect; b – the Festivalnaya variety, degree of effect – 3.0 points.

During the research (2018–2022), in the conditions of Michurinsk, Tambov region, there were both relatively favorable years for the S. macularis (2018, 2019 and 2020) and unfavorable years for the pathogen (2021 and 2022). Under favorable conditions, the degree of strawberry genotypes affected by powdery mildew varied in the range from 0 to 4 points; under conditions unfavorable for the pathogen, plants affected did not exceed 1 point (Fig. 1, Table 2).

**Table 2.**
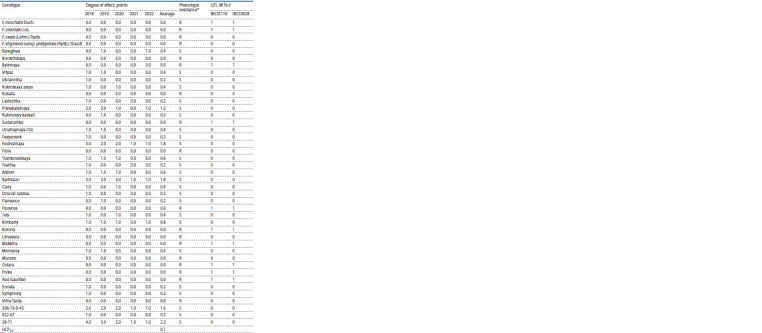
Powdery mildew effect on the studied strawberry genotypes in the conditions of Michurinsk, Tambov region (2018–2022), and the presence of DNA markers for the 08 To-f resistance locus R – resistance (no plants affected); S – susceptibility (affected plants were noted); 1 – target amplicon of DNA marker is present; 0 – target amplicon of DNA marker is absent

Analysis of variance of the obtained results showed a statistically significant influence on the manifestation of the “powdery mildew resistance” trait of both the genotype and the prevailing weather conditions (Table 3).

**Table 3.**
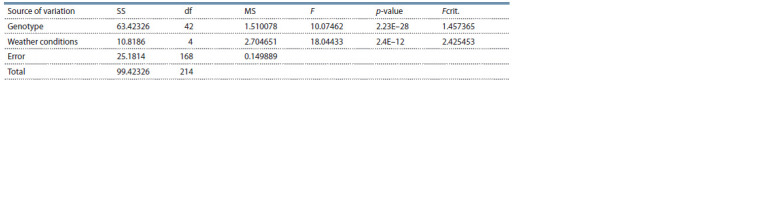
ANOVA test of powdery mildew effect on strawberry genotypes over the years of research Notе. SS – sum of squared deviations; df – degrees of freedom; MS – mean squares; F – F-test (fact. value); p-value – significance of the results; Fcrit. – F-test (crit. value).

At the same time, the predominance of the influence of environmental conditions over the genotype in the formation of the “powdery mildew resistance” trait was noted. The heritability coefficient (H 2) was 34.8 %. The low contribution of genetic variance to the total phenotypic variation of a trait is explained by weather and climatic conditions: out of five years of research, two years (2021 and 2022) were unfavorable for S. macularis, and as a result phenotypic differences between resistant and susceptible strawberry varieties did not appear. Taking into account only the years that were relatively favorable for the pathogen (2018, 2019 and 2020), when the differences between strawberry varieties were more contrasting, the contribution of the genotype to the formation of the trait was 50.7 %.

Variation in the heritability coefficient of the powdery mildew resistance locus depending on the degree of infectious load is also described by other researchers. In particular, it is noted that low phenotypic variability of a trait reduces the calculated heritability coefficient (Kennedy et al., 2014).

Most of the studied strawberry collection (88.4 % forms), including all analyzed wild species of the genus Fragaria, were characterized by high-level resistance to S. macularis – the degree of plants affected did not exceed 1.0 points. It should be noted that in the strawberry variety Sonata, which has resistance loci FxaPMR5b, FxaPMR7A, FxaPMR7X2 (Sargent et al., 2019), the degree of effect over the years of research was on average 0.2 points, and in some years – 1.0 points. In addition, the strawberry variety Clery, obtained by hybridization of the powdery mildew-resistant variety Sweet Charlie (Liu et al., 2012), in some years was characterized by leaf effect of 1 point (the average affected score for 2018–2022 was 0.4).

Over the years of research, 18 out of 43 analyzed strawberry genotypes (41.9 % of the total number of forms) were characterized by the absence of powdery mildew effect. Among the strawberry varieties, the absence of S. macularis effect was detected in 35.9 % genotypes. Among the strawberry varieties of Russian breeding, the absence of S. macularis effect was detected in 29.4 % genotypes, among the foreign strawberry varieties – in 47.4 % forms. At the same time, the differences in the distribution of the “powdery mildew resistance” trait in the samples of Russian and foreign strawberry varieties were statistically insignificant (at a significance level of p ≤ 0.05 tfact = 0.4 ≤ tst = 4.3).

The wild species F. moschata, F. orientalis, F. ovalis and F. virginiana subsp. platypetala, strawberry varieties Borovitskaya, Bylinnaya, Kubata, Sudarushka and Flora (Russian breeding), and Florence, Korona, Limalexia, Malwina, Murano, Ostara, Polka, Red Gauntlet and Vima Tarda (foreign breeding) are resistant to S. macularis (no plants affected over the years of research). The presence of populations with a high level of powdery mildew resistance in many wild strawberry species (F. ovalis, F. virginiana, F. chiloensis, etc.) is confirmed by literature data. At the same time, the ecological and geographical disunity of the habitats of these species suggests the presence of different mechanisms of plant resistance to S. macularis (Kennedy et al., 2013).

It should also be noted that strawberry genotypes that are resistant to powdery mildew in the Tambov region can be affected by the pathogen in other regions. For example, in the conditions of the Altai Territory (Western Siberia), strawberry varieties Korona and Polka were affected by 1.0–1.5 points, and the variety Bylinnaya – by up to 2.5 points (Stolnikova, Kolesnikova, 2017). The results obtained are explained by the presence of physiological races of S. macularis that are specific to different regions.

**Fig. 2.**
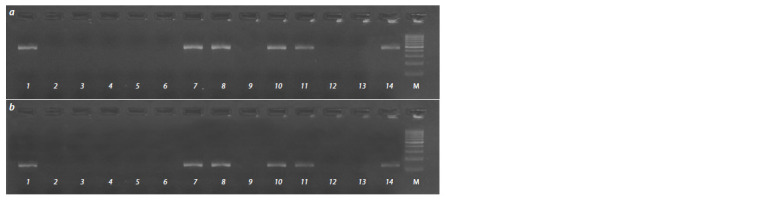
Electrophoresis profile of markers IB535110 (a) and IB533828 (b) of 14 out of 43 analyzed strawberry genotypes 1 – Red Gauntlet; 2 – Symphony; 3 – Bereginya; 4 – Izbrannitsa; 5 – Limalexia; 6 – Monterey; 7 – Bylinnaya; 8 – Korona; 9 – Barlidaun; 10 – Sudarushka; 11 – Polka; 12 – F. ovalis; 13 – F. virginiana subsp. Platypetala; 14 – F. moschata. М – molecular weight marker

To identify genetic factors of resistance, molecular genetic screening of the analyzed collection of strawberry genotypes was carried out using DNA markers IB535110 and IB533828, linked to the 08 To-f powdery mildew resistance locus. Markers IB535110 and IB533828 were identified in 10 forms out of 43, which is 23.2 %. The results obtained for markers IB535110 and IB533828 are characterized by 100 % agreement with the phenotype assessment. An example of identification is shown in Fig. 2; the results are shown in Table 2.

Among the analyzed wild strawberries, the 08 To-f locus was identified in F. moschata and F. orientalis. At the same time, the selected strawberry form 298-19-9-43 (three-species hybrid, second backcross generation from hybridization of F. moschata and F. orientalis) QTL 08 To-f apparently did not inherit it from the original species, since the average degree of powdery mildew affect over the years of research was 1.6 points, the maximum was 2.0 points. Among the strawberry varieties, the proportion of genotypes with an identified powdery mildew resistance locus was 22.2 % (8 varieties out of 36). Among the analyzed Russian strawberry varieties, QTL 08 To-f is present in two forms (Bylinnaya and Sudarushka) out of 17, which is 11.7 %. Among the analyzed foreign strawberry varieties, QTL 08 To-f is present in six genotypes (Florence, Korona, Malwina, Ostara, Polka and Red Gauntlet) out of 19, which is 31.6 %. It should also be noted that in the strawberry variety Sonata, in which the FxaPMR5b, FxaPMR7A and FxaPMR7X2 loci were identified (Sargent et al., 2019), the 08 To-f locus was not identified (markers IB535110 and IB533828 are absent). The obtained results confirm that the powdery mildew resistance of the Sonata variety is determined by QTL identified earlier.

Based on a BLAST analysis of the sequences of the used markers, it was suggested that the loci FxaPMR7A and 08 To-f are orthologous (Sargent et al., 2019). For the strawberry variety Red Gauntlet, other QTLs were also mapped on chromosomes 2A, 4B, 6D, 7C and 7D (Cockerton et al., 2018). Therefore, in the Red Gauntlet variety, phenotypic resistance can be determined by the cumulative effect of several QTLs, and additional studies are necessary to compare the results obtained and clarify the number of identified QTLs and their location.

Analysis of the origin of the strawberry varieties, for which amplification fragments with markers IB535110 and IB533828 were obtained, indicates that the Florence and Ostara varieties were obtained using the Red Gauntlet variety, which, according to the data obtained, is the source of QTL 08 To-f. The strawberry variety Korona was obtained in the crossing combination Tamella × Unduka, the Polka variety was obtained in the hybrid combination Unduka × Sivetta, and therefore the source of QTL 08 To-f for these forms is presumably the Unduka variety. To clarify, it is necessary to analyze the original parental strawberry forms for the presence of diagnostic markers IB535110 and IB533828

It should also be noted that using the strawberry varieties Red Gauntlet and Unduka, varieties Borovitskaya (Nadezhda × Red Gauntlet), Tsaritsa (Venta × Red Gauntlet) and Bereginya (Solovushka × Unduka) were obtained, which, according to the results of a molecular genetic analysis, do not have the 08 To-f powdery mildew resistance locus. The strawberry variety Bylinnaya was obtained in the crossing combination Persikovaya × Seyanets VIR-228613; for the original parental forms, there is no data on the presence of QTL 08 To-f. For the strawberry variety Sudarushka, the initial parental forms are Festivalnaya and Roxana. The strawberry variety Festivalnaya was affected by powdery mildew over the years of research by an average of 1.8, with the effect varying over the years from 1.0 points to 3.0 points; the variety does not have markers IB535110 and IB533828. Therefore, the source of QTL 08 To-f for the strawberry variety Sudarushka is presumably the Roxana variety. 

A comparison of the results of molecular genetic analysis and phenotypic assessment of resistance to powdery mildew showed that all strawberry genotypes with identified markers IB535110 and IB533828 in the conditions of Michurinsk, Tambov region, are characterized by field resistance to S. macularis (no plants affected over the years of research). Thus, QTL 08 To-f is a promising component of the genetic determinant of resistance to local races of S. macularis, and the strawberry varieties Bylinnaya, Sudarushka, Florence, Korona, Malwina, Ostara, Polka and Red Gauntlet as well as the wild species F. moschata and F. orientalis are valuable initial forms that can be used in breeding programs to create pathogen-resistant strawberry genotypes. At the same time, to reduce research time, financial and labor resources, it is permissible to use one of the two DNA markers. The disadvantage of the diagnostic markers used is the presence of only one target amplicon, and therefore it is possible to obtain false negative results due to PCR inhibition. To exclude false negative results, it is necessary to conduct a preliminary assessment of the quality of the extracted total strawberry DNA.

In addition, according to previous studies, the strawberry variety Bylinnaya is characterized by the presence of the Rpf1 red stele root rot resistance gene (the causative agent is Phytophthora fragariae var. fragariae Hickman) (Lyzhin, Luk’yanchuk, 2020), and the strawberry variety Sudarushka is characterized by the presence of the Rca2 anthracnose resistance gene (the causative agent is Colletotrichum acutatum J.H. Simmonds) (Lyzhin et al., 2019). Therefore, these strawberry varieties are complex sources of resistance alleles to fungal pathogens

It should also be noted that some of the studied strawberry genotypes (wild species F. ovalis and F. virginiana subsp. platypetala; strawberry varieties Borovitskaya, Kubata, Flora, Limalexia, Murano and Vima Tarda), in which QTL 08 To-f is absent, were not affected by powdery mildew over the years of research

The dependence of phenotypic resistance to S. macularis on the presence of QTL 08 To-f in the genotype in the analyzed strawberry forms is described by the regression equation y = 0.758x + 0.242. Testing the significance of the regression model using Fisher’s F test showed that at a significance level of 0.05, the null hypothesis about the absence of dependence between the variables is refuted (Table 4).

**Table 4.**
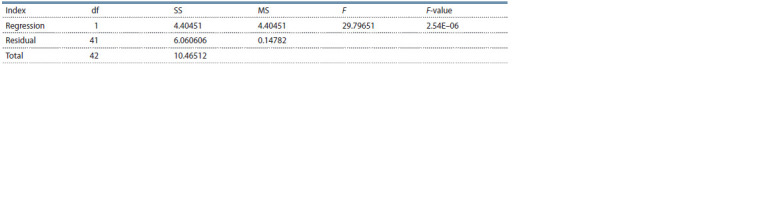
Results of regression analysis of the dependence of strawberry phenotypic resistance to powdery mildew on the presence of the 08 To-f locus Notе. df – degrees of freedom; SS – sum of squared deviations; MS – mean squares; F – F-test (fact. value); F-value – significance of the results.

The degree of correlation between the presence of markers IB535110 and IB533828 and phenotypic resistance (no powdery mildew infection) was 0.649, which on the Chaddock scale corresponds to a perceptible communication between the traits (0.5 < rxy < 0.7). The coefficient of determination (R2), showing the contribution of the studied locus to the trait manifestation, is equal to 0.420, that is, in 42 % of cases, phenotypic resistance is determined by the presence of QTL 08 To-f, and in 58 % of cases, it is affected by other factors. According to literature data, the coefficient of determination (R2) for some resistance loci identified for powdery mildew varied from 0.16 to 0.57 (Cockerton et al., 2018).

The obtained results indicate the presence of additional genetic determinants of resistance to S. macularis in these strawberry forms. In this regard, identification of powdery mildew-resistant genotypes in the strawberry hybrid progeny using diagnostic DNA markers IB535110 and IB533828 is possible if the parental forms have QTL 08 To-f. In other cases, strawberry resistance to S. macularis may be controlled by other genetic factors and, therefore, the use of markers IB535110 and IB533828 is inappropriate.

## Conclusion

Thus, diagnostic DNA markers IB535110 and IB533828 make it possible to reliably identify the 08 To-f powdery mildew resistance locus in the strawberry genoplasm and can be used in marker-assisted strawberry assortment improvement programs. Promising sources of resistance to S. macularis, according to the results of molecular genetic analysis, are the wild species F. moschata and F. orientalis, and strawberry varieties Bylinnaya and Sudarushka (Russian breeding), Florence, Korona, Malwina, Ostara, Polka and Red Gauntlet (foreign breeding).

## Conflict of interest

The authors declare no conflict of interest.
